# Association of APOA5 rs662799 and rs3135506 polymorphisms with arterial hypertension in Moroccan patients

**DOI:** 10.1186/1476-511X-13-60

**Published:** 2014-04-01

**Authors:** Sanaa Ouatou, Maria Ajjemami, Hicham Charoute, Hajar Sefri, Noreddine Ghalim, Houria Rhaissi, Houda Benrahma, Abdelhamid Barakat, Hassan Rouba

**Affiliations:** 1 Laboratoire de Génétique Moléculaire et Humaine, Département de Recherche Scientifique, Institut Pasteur du Maroc, 1, Place Louis Pasteur, 20360 Casablanca, Morocco; 2 Laboratoire de Biochimie, Centre de Biologie Médicale, Institut Pasteur du Maroc, 1, Place Louis Pasteur, 20360 Casablanca, Morocco; 3 Laboratoire de Physiologie et Génétique Moléculaire, Faculté des sciences Ben M’Sik, Université Hassan II, Mohammedia, Morocco

**Keywords:** APOA5 gene, Polymorphisms, Haplotypes, Arterial hypertension, Morocco

## Abstract

**Background:**

The goal of the study is to investigate the association between the APOA5 polymorphisms and haplotypes with Arterial Hypertension (AHT) in Moroccan patients.

**Methods:**

The study was performed in 283 subjects, 149 patients with AHT and 134 controls. All subjects were genotyped for the APOA5 -1131 T > C (rs662799), 56C > G (rs3135506) and c.553G > T (rs2075291) polymorphisms.

**Results:**

There was a strong association between -1131 T > C and 56C > G polymorphisms with AHT. The -1131 T > C and 56C > G polymorphisms were significantly associated with increased systolic blood pressure (SBP) and triglycerides (TG) levels. There were 4 haplotypes with a frequency higher than 5%, constructed from APOA5 polymorphisms, with the following order: -1131 T > C, 56C > G and c.553G > T. Haplotype H1 (TCG) was associated with decreased risk of AHT, whereas the haplotypes H2 (CCG) and H4 (CGG) were significantly associated with an increased risk of AHT. Carriers of H1 haplotype had a lower SBP and DBP and TG. In contrast, significant elevated SBP, DBP and TG were found in H4 haplotypes carriers.

**Conclusions:**

Our data demonstrate for the first time that several common SNPs in the *APOA5* gene and their haplotypes are closely associated with modifications of blood pressure and serum lipid parameters in the AHT patient.

## Background

Arterial hypertension (AHT) is one of major public health problem in the world, it will affect more than 1.56 billion adults worldwide in 2025 [1]. Hypertensive individuals have higher risk to develop coronary artery disease (CAD), cerebrovascular disease and heart failure than normotensive persons [2]. Effectively, The American Heart Association reported that the hypertension and dyslipidemia are one of the main risk factor for development of CAD [3,4]. AHT is a polygenic and multifactorial disease resulting from combination between genetics and environment factors.

Apolipoprotein A5 (*APOA5*) gene, related to the metabolism of triglycerides in several different ethnic groups [5]. It locates on chromosome 11q23, and forms a cluster with *APOA4, APOC3* and *APOA1* genes. In mice, invalidation and over-expression of *APOA5* result in increased and decreased triglyceride levels, respectively by controlling the activity of lipoprotein lipase (LPL) [1]. *APOA5* codes for a protein of 366 amino acids which function is to modulate intracellular hepatic VLDL synthesis. The most studied *APOA5* SNP was the 56C > G (S19W, rs 3135506), because it is associated with increased TG levels [6,7]. Indeed, numerous studies in different ethnic populations have shown significant associations between two minor *APOA5* haplotypes, APOA5*2 and APOA5*3, and elevated plasma triglyceride levels [5,8]. It is estimated that 53% of Hispanics, 35% of African-Americans, and 24% of Caucasians carry at least one of these two haplotypes [6], thus suggesting that these haplotypes are common risk factors for atherosclerosis.

In this study, we investigated the association of the APOA5 polymorphisms and haplotypes with AHT in Moroccan patients.

## Methods

### Study population

Our study concerned 283 Moroccan adult volunteers (177 women and 106 men), 149 patients diagnosed as hypertensive by medical corps (systolic blood pressure >140 mmHg and/or diastolic blood pressure >90 mmHg, and treated by antihypertensive drugs) and 134 controls (non smoking healthy persons with normal lipid levels and normal blood pressure, and women were non-pregnant and non-breast-feeding). At enrolment in the Medical Biology Center of Pasteur Institute of Morocco in Casablanca, they completed a health and lifestyle questionnaire including social demographics characteristics, medical history, medications intake and lifestyle factors as tobacco consumption, physical activity and alcohol intake. The clinical examination consisted of questionnaire and a physical examination: age, gender, geographical origin, family history, body mass index, smoking habits, the presence of hypertension, diabetes, hypercholesterolemia or cocaine use, and levels of physical activity and alcohol consumption performed at the Pasteur Institute in Casablanca. All subjects (patients and controls groups) included in this study were from different geographic and ethnic backgrounds from Morocco.

### Ethics statement

We obtained written informed consent from each subject and the research protocol was approved by the committee on research ethics of Pasteur Institute of Morocco.

### Blood pressure measurement

Arterial pressure was measured by the auscultatory method using a stethoscope and a sphygmomanometer. All measurements were performed by nurse in the Medical Biology Center of Pasteur Institute of Morocco in Casablanca.

### Biochemical measurements

The blood was collected in two tubes (EDTA and dry tubes) from subject after 12 hours overnight fasting. The samples were centrifuged and stored at -20°C. Glycemia, total cholesterol (TC), Triglycerides (TG) and High-Density Lipoprotein Cholesterol (HDL-C) levels were determined using the VITROS (5.1 FS Chemistery System). Low-density Lipoprotein Cholesterol (LDL-C) level was calculated according to the Friedwald’s formula. All biochemical measurements were performed in the Biochemistry Laboratory of the Medical Biology Center in Pasteur Institute of Morocco.

### Molecular analysis: genotyping APOA5 polymorphisms

Genomic DNA was extracted from peripheral leukocytes by standard methods including proteinase K digestion, followed by phenol–chloroform extraction and ethanol precipitation. The 56C > G and -1131 T > C polymorphisms were determined by PCR-RFLP analysis. All PCR were performed in a Biometra thermal cycler, using Taq Polymerase (Bioline). A fragment of 157 bp including the 56C > G polymorphism was amplified using two oligonucleotides, forward: 5′- GGC TCT TCT TTC AGG TGG GTCBTCCG -3′reverse: 5′- GCC TTT CCG TGC CTG GGT GGT -3′ [9]. The PCR conditions were an initial denaturing at 96°C for 5 min, followed by 30 cycles of 96°C for 30 s, 64°C for 30 s, 72°C for 45 s, and a final extension of 72°C for 10 min. The PCR products were digested for 2 hours at 65°C with *TaqI* restriction enzyme: the C56 allele presents a *TaqI* restriction site which is suppressed in the 56G allele. Genotyping for -1131 T > C was performed with the following primers: Forward: 5′- CCC CAG GAA CTG GAG CGA AATT-3′, reverse 5′- TTC AAG CAG AGG GAA GCC TGTA-3′. The PCR conditions were an initial denaturing at 96°C for 5 min, followed by 32 cycles of 95°C for 30 s, 55°C for 30 s, 72°C for 30 s, and a final extension of 72°C for 10 min. The PCR products were digested with Mse I. A fragment of 138 bp of the c.553G > T polymorphism was amplified using two oligonucleotides. The PCR conditions were an initial denaturing at 96°C for 5 min, followed by 35 cycles of 96°C for 30 s, 63°C for 30 s, 72°C for 45 s, and a final extension of 72°C for 10 min. The PCR products were digested for overnight at 37°C with *HaeIII* restriction enzyme. All molecular analyses were performed in the Human Genetic Laboratory in Pasteur Institute of Morocco.

### Statistical analysis

Clinical and biochemical data were expressed as means ± standard deviation (SD). Student’s t test was applied for comparison of quantitative traits that follow a normal distribution. Otherwise, we used Manne-Whitney test. Chi-square test and logistic regression analysis were performed to test the association between Arterial Hypertension and APOA5 genotypes and haplotypes. Logistic regression analysis was adjusted by age and gender. A P value of less than 0.05 was considered statistically significant. All statistical analyses were performed using STATA software, version 11.0. The P-values were corrected with the Bonferroni correction by multiplying with the number of comparisons. For haplotype frequencies estimation and comparison we used the PLINK software, version 1.07. All haplotypes with frequencies less than 5% were ignored in analysis. Linkage disequilibrium between each pair of APOA5 polymorphisms was estimated using Haploview software, version 4.2.

## Results

### Characteristics of controls and patients

Clinical characteristics and lipid parameters of AHT patients and controls are presented in Table 1. As expected, age, triglyceridemia (TG), LDL, total cholesterol (TC), Glycemia (Gly), CT/HDL ratio, Sbp and Dbp were significantly different between AHT patients and controls. In addition, HDL/LDL ratio was significantly elevated in controls. There was no significant difference in body mass index (BMI) and HDL between both groups.

**Table 1:** Clinical and biochemical characteristics of controls and patients with AHT

	**Controls (n = 134)**	**Patients (n = 149)**	**P-value**
Age (years)	50.78 ± 12.08	59.76 ± 11.65	<0.0001
Sbp (mmgHg)	121.69 ± 6.77	153.09 ± 19.19	<0.0001
Dbp (mmgHg)	77.49 ± 7.97	85.97 ± 10.91	<0.0001
BMI (kg/m^2^)	26.49 ± 4.11	27.42 ± 4.63	0.0739
TG (g/L)	0.98 ± 0.31	1.65 ± 0.85	<0.0001
LDL (g/L)	1.15 ± 0.28	1.34 ± 0.45	<0.0001
HDL (g/L)	0.51 ± 0.13	0.50 ± 0.17	0.5042
Gly (g/L)	0.91 ± 0.14	1.23 ± 0.57	<0.0001
TC (g/L)	1.88 ± 0.28	2.12 ± 0.51	<0.0001
TC/HDL	3.90 ± 0.94	4.64 ± 1.61	<0.0001
HDL/LDL	0.48 ± 0.24	0.43 ± 0.32	0.0002

BMI: bodymass index, TC: Serum total cholesterol, TG: Triglycerides, HDL: High-Density Lipoprotein Cholesterol, LDL: Low-density Lipoprotein Cholesterol, Sbp systolic blood pressure, Dbp diastolic blood pressure.

### Genotype frequency and linkage disequilibrium

The -1131 T > C, 56C > G SNPs had a strong association with AHT in all genetic models, the p-values remained significant after Bonferroni correction**.** The c.553G > T polymorphism had a significant association with AHT in co-dominant and dominant models, but this association was lost after Bonferroni correction (Table 2).

**Table 2:** **Genotypic distribution of *APOA5 *** **polymorphisms and statistic comparison between AHT subjects and controls**

**SNP**	**Model**	**Controls**	**Patients**	**OR (95% CI)**	**P**	**Pc**
-1131 T > C						
	Codominant					
	TT	128 (95.5%)	97 (65.1%)	1.00		
	TC	2 (1.5%)	20 (13.4%)	11.16 (2.46-50.69)	0.002	0.006^*^
	CC	4 (3%)	32 (21.5%)	14.20 (4.50-44.77)	<0.0001	<0.0001^*^
	Dominant					
	TT	128 (95.5%)	97 (65.1%)	1.00		
	TC/CC	6 (4.5%)	52 (34.9%)	13.20 (5.14-33.91)	<0.0001	<0.0001^*^
	Recessive					
	TT/TC	130 (97%)	117 (78.5%)	1.00		
	CC	4 (3%)	32 (21.5%)	12.35 (3.91-39.05)	<0.0001	<0.0001^*^
56C > G						
	Codominant					
	CC	117 (87.3%)	106 (71.1%)	1.00		
	CG	15 (11.2%)	21 (14.1%)	1.54 (0.72-3.29)	0.269	NS
	GG	2 (1.5%)	22 (14.8%)	13.75 (3.01-62.75)	0.001	0.003^*^
	Dominant					
	CC	117 (87.3%)	106 (71.1%)	1.00		
	CG/GG	17 (12.7%)	43 (28.9%)	2.90 (1.49-5.63)	0.002	0.006^*^
	Recessive					
	CC/CG	132 (98.5%)	127 (85.2%)	1.00		
	GG	2 (1.5%)	22 (14.8%)	12.77 (2.82-57.73)	0.001	0.003^*^
c.553G > T						
	Codominant					
	GG	130 (97%)	132 (88.6%)	1.00		
	GT	3 (2.2%)	15 (10.1%)	4.72 (1.28-17.35)	0.020	NS
	TT	1 (0.8%)	2 (1.3%)	1.64 (0.14-19.78)	0.695	NS
	Dominant					
	GG	130 (97%)	132 (88.6%)	1.00		
	GT/TT	4 (3%)	17 (11.4%)	3.92 (1.23-12.43)	0.02	NS
	Recessive					
	GG/GT	133 (99.2%)	147 (98.7%)	1.00		
	TT	1 (0.8%)	2 (1.3%)	1.47 (0.12-17.70)	0.763	NS

NS: Not Significant.

Pc: P value corrected for multiple comparisons (Bonferroni correction).

*P values remains significant after Bonferroni correction.

**Figure 1:**
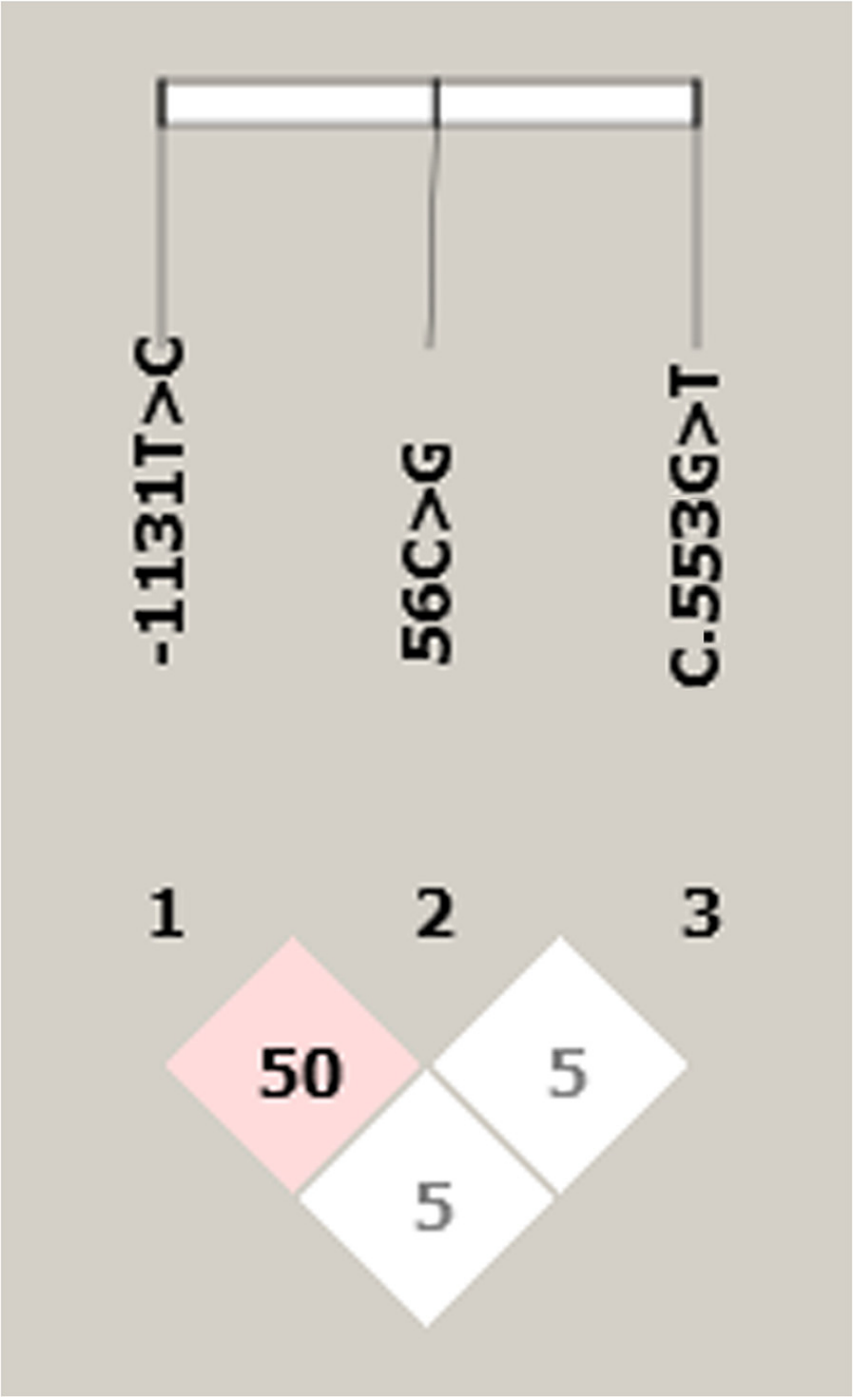
The linkage disequilibrium (LD) between the three APOA5 SNPs.

To determine the extent of linkage disequilibrium (LD) among the three polymorphisms, standardized LD coefficient D’ was calculated for all pairs of polymorphisms. Figure 1 shows that with the exception of -1131 T > C and 56C > G polymorphisms which were in strong linkage disequilibrium (D’ = 50), other polymorphisms were not in linkage disequilibrium (Figure 1).

### Comparisons of clinical and biochemical parameters between APOA5 genotypes

We compared biological and clinical traits between APOA5 genotypes under dominant model for all patients and controls combined. The APOA5 -1131 T > C polymorphism was significantly associated with increased levels of systolic blood pressure, diastolic blood pressure and triglycerides (all P values <0.0001). We found significantly elevated systolic blood pressure (P = 0.0001) and triglycerides levels (P < 0.0001) in association with APOA5 56C > G polymorphism. No Significant association was observed for APOA5 c.553G > T polymorphism (Table 3).

**Table 3:** **Association between *APOA5 *** **genotype variants and clinical and biochemical parameters**

	**APOA5 -1131 T > C**			**APOA5 56C > G**			**APOA5 c.553G > T**		
	**TT**	**TC + CC**	**P-value**	**CC**	**CG + GG**	**P-value**	**GG**	**GT + TT**	**P-value**
Patient (number)	97	52		106	43		132	17	
Controls (number)	128	6		117	17		130	4	
Age (years)	54.94 ± 12.62	57.72 ± 12.68	0.1353	55.13 ± 12.62	56.90 ± 12.82	0.3386	55.24 ± 12.84	58.81 ± 9.87	0.215
Sbp (mmgHg)	133.67 ± 19.71	155.86 ± 18.94	<0.0001**	135.32 ± 20.27	148.98 ± 22.62	0.0001**	137.82 ± 21.15	143.24 ± 25.40	0.2495
Dbp (mmgHg)	80.57 ± 10.60	87.33 ± 8.24	<0.0001**	81.27 ± 9.43	84.48 ± 13.59	0.0355*	82.13 ± 10.33	79.81 ± 12.60	0.3319
BMI (kg/m^2^)	26.82 ± 4.21	27.58 ± 5.11	0.2428	26.79 ± 4.21	27.68 ± 5.06	0.1676	27.09 ± 4.41	25.63 ± 4.30	0.1446
TG (g/L)	1.23 ± 0.63	1.76 ± 0.93	<0.0001**	1.23 ± 0.64	1.75 ± 0.91	<0.0001**	1.32 ± 0.74	1.53 ± 0.65	0.0564
LDL (g/L)	1.22 ± 0.37	1.37 ± 0.44	0.0101*	1.24 ± 0.38	1.31 ± 0.43	0.1889	1.25 ± 0.39	1.30 ± 0.43	0.5751
HDL (g/L)	0.51 ± 0.17	0.47 ± 0.11	0.0631	0.51 ± 0.16	0.47 ± 0.12	0.0746	0.50 ± 0.16	0.53 ± 0.16	0.3976
Gly (g/L)	1.06 ± 0.46	1.13 ± 0.43	0.0582	1.09 ± 0.48	1.04 ± 0.29	0.9913	1.06 ± 0.43	1.33 ± 0.65	0.0668
TC (g/L)	1.97 ± 0.41	2.15 ± 0.52	0.0045*	1.98 ± 0.41	2.10 ± 0.51	0.0627	2.00 ± 0.44	2.13 ± 0.42	0.1688

*P values not significant after Bonferroni correction.

**P values remains significant after Bonferroni correction.

### APOA5 haplotype analysis

To examine the combined effect of three variants (in the order of - 1131 T > C, 56C > G and c.553G > T polymorphisms of APOA5) in the APOA5 gene. There were 4 haplotypes identified in the APOA5gene in our population, with frequencies greater than 5% (Table 4). Results of the logistic regression analysis suggested that the haplotype H1 (APOA5*1) has a protective effect against AHT. Two haplotypes confers significant susceptibility to AHT; haplotype H2 (APOA5*2) and haplotype H4. No significant association was observed between the haplotype H3 (APOA*3) and AHT. The frequencies of the four haplotypes are listed in Table 4. Analysis of the interaction between haplotypes, Serum lipid parameters and environment factors showed that the H1 haplotype was associated with lower systolic blood pressure (SBP), diastolic blood pressure (DBP) and total plasma (TG). The H2 haplotype was significantly associated with increased SBP. Carriers of the H4 haplotype had a significant increased SBP, DBP and TG. No significant difference was detected between carriers and non-carriers of H3 haplotype. Age, BMI, LDL, HDL and glycemia were also analyzed but no significant difference was found in these parameters among the four haplotypes (Table 5).

**Table 4:** Association analysis of haplotypes derived from polymorphic sites using genotype data

**Haplotype**	**-1131 T > C**	**56C > G**	**c.553G > T**	**Frequency**		**OR (95% CI)**	**P**	**Pc**
				**Controls**	**Cases**			
H1	T	C	G	0.8970	0.6543	0.338 (0.226-0.504)	<0.0001	<0.0001
H2	C	C	G	0.0307	0.1307	3.07 (1.49-6.31)	0.0005	0.002
H3	T	G	G	0.0649	0.0625	0.809 (0.395-1.66)	0.563	NS
H4	C	G	G	0.0073	0.1525	12.46 (2.93-53 )	<0.0001	<0.0001

NS: Not Significant.

Pc: P value corrected for multiple comparisons (Bonferroni correction).

**Table 5:** **Association between *APOA5 *** **haplotypes and clinical and biochemical parameters**

	**Haplotype H1 (TCG)**			**Haplotype H2 (CCG)**			**Haplotype H3 (TGG)**			**Haplotype H4 (CGG)**		
	**Present**	**Not present**	**P value**	**Present**	**Not present**	**P value**	**Present**	**Not present**	**P value**	**Present**	**Not present**	**P value**
Age (years)	55.10 ± 12.36	57.52 ± 14.00	0.2276	56.97 ± 13.12	55.33 ± 12.62	0.4976	57.03 ± 14.38	55.33 ± 12.46	0.4865	57.50 ± 12.69	55.25 ± 12.66	0.3458
Sbp (mmgHg)	134.79 ± 19.84	155.00 ± 21.55	<0.0001**	152.55 ± 17.19	136.46 ± 21.33	<0.0001**	139.10 ± 21.19	138.11 ± 21.56	0.7795	157.56 ± 21.21	135.75 ± 20.27	<0.0001**
Dbp (mmgHg)	81.03 ± 10.41	86.48 ± 9.88	0.001**	86.35 ± 8.47	81.41 ± 10.62	0.0132*	81.40 ± 16.88	82.02 ± 9.52	0.7606	87.88 ± 8.59	81.20 ± 10.50	0.0006**
BMI (kg/m2)	26.85 ± 4.30	27.64 ± 4.90	0.2596	26.50 ± 4.67	27.04 ± 4.38	0.5181	27.11 ± 4.88	26.97 ± 4.36	0.8684	28.12 ± 5.56	26.83 ± 4.23	0.1195
TG (g/L)	1.24 ± 0.65	1.81 ± 0.92	<0.0001**	1.38 ± 0.69	1.33 ± 0.74	0.497	1.61 ± 1.00	1.31 ± 0.69	0.1055	1.99 ± 0.98	1.25 ± 0.65	<0.0001**
LDL(g/L)	1.23 ± 0.37	1.36 ± 0.47	0.0402*	1.42 ± 0.46	1.23 ± 0.38	0.0107*	1.24 ± 0.43	1.25 ± 0.39	0.8132	1.36 ± 0.45	1.24 ± 0.38	0.0883
HDL(g/L)	0.51 ± 0.16	0.45 ± 0.10	0.0061*	0.48 ± 0.10	0.50 ± 0.16	0.4389	0.47 ± 0.14	0.51 ± 0.16	0.2057	0.47 ± 0.12	0.51 ± 0.16	0.1585
Gly (g/L)	1.07 ± 0.46	1.12 ± 0.39	0.2406	1.16 ± 0.49	1.07 ± 0.44	0.1619	0.99 ± 0.21	1.09 ± 0.47	0.6938	1.08 ± 0.33	1.08 ± 0.46	0.5571
TC (g/L)	1.98 ± 0.41	2.15 ± 0.54	0.0122*	2.15 ± 0.53	1.99 ± 0.42	0.0604	2.00 ± 0.48	2.01 ± 0.43	0.8825	2.18 ± 0.54	1.98 ± 0.42	0.0162*

*P values not significant after Bonferroni correction.

**P values remains significant after Bonferroni correction.

## Discussion

Many evidences were reported showing that apolipoprotein A1/C3/A4/A5 gene cluster is associated with premature coronary artery disease [10] and serum lipid levels [10]. Recent findings indicate that APOA5 could also influence cholesterol homeostasis and probably could play a role in hypertriglyceridemia associated with diabetes and inflammation [11].

The APOA5 polymorphisms were also identified to be implicated in regulation of blood pressure and in the development of hypertension in Japanese population [12].

Our data demonstrate that the 56C > G SNP has a significant influence on blood pressure and triglyceride levels. This variant had not previously been investigated in Moroccan populations, although several different studies of other populations are available, supporting the importance of our study. The frequency of the 56G rare allele detected in Moroccan patients (22%) is higher than that of European populations. Indeed, studies carried out with North Americans and Europeans found that the 56G allele frequency is around 6% [6,7,13], whereas its frequency in North Americans of Hispanic ancestry is around 15% [14]. Ruiz- Narváez registered a value of 10.2% for the 56G allele in Costa Rica, while other populations exhibited lower frequencies in the same study (Caucasians 6%, African-Americans 7%) [15]. In addition to these studies, in 2003, Lai et al. investigated the frequency of the same polymorphism in people resident in Singapore [8]. The Chinese, Malay and Indian populations living in this region exhibited extremely low frequencies of the 56G polymorphism (0.1%, 1% and 3%, respectively). The 56G allele frequency varies between 0.1% in a Chinese population [8] and 15.8% in Hispanic males [16].

*APOA5* gene codes for an apolipoprotein involved in the regulation of LPL activity [17,18]. ApoAV protein could facilitate the interaction between TG rich lipoproteins and proteoglycan-bound LPL. Transgenic mice models also suggested that apoAV might inhibit VLDL hepatic production but these findings were not confirmed in lipoprotein kinetic studies [19].

Although the association of 56C > G polymorphism with lipid profile was already shown in various healthy populations [20], their impact on AHT dyslipidemia remains undocumented. In another study, this polymorphism was associated with higher TG and lower HDLc in diabetic patients, but only for Indian-Asian carriers [21]. There was no report of the impact of this polymorphism on history of dyslipidemia in AHT. Nevertheless, an increased 56G allele frequency was previously reported in non diabetic patients with severe hypertriglyceridemia [22,23]. The association between the 56G allele and increased triglycerides has already been documented in other populations, such as the North American and Northern Irish populations [24,25]. The results of the present study confirm the relationship between *APOA5*56C > G polymorphism and plasma triglycerides, as carriers of the 56G variant were associated strongly with triglyceride levels. In addition to elevated triglyceride levels, the APOA5 56G was associated with higher systolic blood pressure relative to 56C carriers. A direct effect of APOA5 on blood pressure regulation is unlikely. In contrast, there is experimental evidence to suggest that chronic hypertriglyceridemia leads to endothelium dysfunction, which is associated with an impaired response to vasodilator stimulation [26] and a subsequent decrease in nitric oxide availability phenomena, which may result in increased blood pressure.

We found a significant difference between patients with AHT and controls regarding the frequency of 56C > G and -1131 T > C genotypes in the additive, dominant and recessive models.

Several studies demonstrated that the presence of polymorphisms in the A1-C3-A4 cluster and other gene loci determines the variability of the postprandial lipoprotein response [27,28]. Recently a gene coding for *APOA5* was identified in this cluster, and this is emerging as a main candidate gene for modulating TG metabolism in humans [6]. Two polymorphisms,-1131 T > C and 56C > G, have been extensively studied and are independently associated with higher TG levels [6,28,29]. Previous studies have shown that plasma TG concentrations were 69% higher in CC subjects than TT subjects with the-1131 T > C polymorphism [8,30] and 20–30% higher in CG than CC subjects with the 56C > G polymorphism [6,31]. Moreno et al. demonstrated that carriers of the -1131C allele (-1131 T > C) displayed a higher plasma TG concentration [32]. However, association studies using haplotypes should increase our ability to detect true associations and interactions.

The *ApoA5* -1131C allele in our study population was similar to that in Chinese (29.9%) [33-35], Singaporean (29.4%) [8], Malays (30.0%) [8], slightly lower than that in Japanese (34.0%) [36,37], but much greater than that of whites (8.0%) [5], Hispanic Americans (13.0%-16.0%) [6,38] or Tunisian (13.0%) [39].The frequency of *ApoA5* c.553G > T allele in this study is extremely low, and is in agreement with that of two previous studies in Chinese (3.97%) [40] and Chinese Taiwanese (4.2-7.2%) [41,42]. The *ApoA5* c.553 T allele has been reported to be absent in Caucasians [43]. The *ApoA5* c.553TT homozygous was similarly detected in our study population in agreement with a previous study [40].

To the best of our knowledge, this study is the first to determine that different haplotypes of *APOA5* gene modulate the systolic, diastolic blood pressure and lipid levels in AHT patients. Thus, our data show markedly higher systolic and diastolic blood pressure in subjects with the *APOA5 H2* and *APOA5 H4* haplotypes, which may explain the higher risk of coronary heart disease associated with the 56G and -1131C alleles [31,44].

In our study, both the *APOA5* H2 and *APOA5* H4 haplotypes were significantly associated with increase in systolic blood pressure. In addition the H4 haplotype was associated with higher DBP and TG levels. In contrast the H1 haplotype showed significant association with lower SBP, DBP and total plasma TG. These results suggest that each of these haplotypes may be associated with different mechanisms that enhance the plasma lipid levels and the risk of atherogenesis.

All these data suggested the importance of *APOA5* in the regulation of plasma triglyceride concentrations. Furthermore, pair wise linkage disequilibrium comparison performed in this study between *APOA5* -1131 T < C and *APOA5* 56C > G demonstrated that they are linked, suggesting cooperation mechanisms for the associations with plasma lipoproteins and related traits. Metabolic syndrome (MetS) is a cluster of disorders which includes visceral obesity, dyslipidaemia, hyperglycaemia, and hypertension [45]. The association of APOA5 polymorphisms with increased risk of metabolic syndrome was showed in several studies [46]. The APOA5 gene plays an important role in regulating triglyceride levels. This regulation may contribute to the association between APOA5 and hypertension showed in this paper, but we cannot exclude other mechanisms.

Our study has same limitations; waist conference wasn’t measured to assess central obesity. In addition, the APOA5 -1131 T > C and c.553G > T polymorphisms were not in Hardy-Weinberg equilibrium.

In summary, we demonstrated that common variants of *APOA5* gene are associated with AHT and contribute to the variation in human plasma TG. Therefore, *APOA5* variant was a significant predictor for high triglyceride risk and the APOA5 haplotypes affected dyslipidemia appreciably among the Moroccan population.

## Conclusion

In conclusion, we demonstrate for the first time that APOA5 polymorphisms and haplotypes have a strong influence on systolic and diastolic blood pressures and triglyceride levels in Moroccan patients with AHT. Our results showed that APOA5 56C > G and -1131 T > C polymorphisms play a major role in elevated risk of developing coronary artery disease due to its association with increased plasma TG.

## Competing interests

The authors declare that they have no competing interests.

## Authors’ contributions

AB conceived, designed and coordinated the study. SO, MA and HS performed the laboratory work. HC and HB carried out statistical analysis. SO, HC and HB wrote the paper. NG, HRh, HRo participated in statistical analyses and interpretation of results. All authors read and approved the final manuscript.
